# Combining lexical and context features for automatic ontology extension

**DOI:** 10.1186/s13326-019-0218-0

**Published:** 2020-01-13

**Authors:** Sara Althubaiti, Şenay Kafkas, Marwa Abdelhakim, Robert Hoehndorf

**Affiliations:** 1 Computational Bioscience Research Center, King Abdullah University of Science and Technology, Thuwal, 23955-6900 Saudi Arabia; 2 Computer, Electrical and Mathematical Sciences and Engineering Division, King Abdullah University of Science and Technology, Thuwal, 23955-6900 Saudi Arabia

**Keywords:** Disease ontology, Embeddings, Neural network

## Abstract

**Background:**

Ontologies are widely used across biology and biomedicine for the annotation of databases. Ontology development is often a manual, time-consuming, and expensive process. Automatic or semi-automatic identification of classes that can be added to an ontology can make ontology development more efficient.

**Results:**

We developed a method that uses machine learning and word embeddings to identify words and phrases that are used to refer to an ontology class in biomedical Europe PMC full-text articles. Once labels and synonyms of a class are known, we use machine learning to identify the super-classes of a class. For this purpose, we identify lexical term variants, use word embeddings to capture context information, and rely on automated reasoning over ontologies to generate features, and we use an artificial neural network as classifier. We demonstrate the utility of our approach in identifying terms that refer to diseases in the Human Disease Ontology and to distinguish between different types of diseases.

**Conclusions:**

Our method is capable of discovering labels that refer to a class in an ontology but are not present in an ontology, and it can identify whether a class should be a subclass of some high-level ontology classes. Our approach can therefore be used for the semi-automatic extension and quality control of ontologies. The algorithm, corpora and evaluation datasets are available at https://github.com/bio-ontology-research-group/ontology-extension.

## Background

The biomedical community has spent significant resources to develop biomedical ontologies which contain and define the basic classes and relations that occur within a domain. Biomedical ontologies are developed by domain experts and are often developed in conjunction with the needs arising in literature-based curation of biological databases.

Manual curation of databases based on literature is a very time-consuming task due to the massive amounts of literature, and automated methods have been developed early on to aid in curation [1]. One of the key tasks in computational support for literature curation is the automatic concept recognition of mentions of ontology classes in text [2]. An ontology class is an intensionally defined entity that has a formal description within an ontology and axioms that determine its relation with other classes [3]. In natural language, multiple terms and phrases can be used to refer to an ontology class [4], and the formal dependencies within an ontology further determine whether a term refers to a class or not (i.e., whether a term refers to a particular class may depend on background knowledge, in particular subclass relations, contained in an ontology). For example, the Disease Ontology (DO) [5] declares *Prediabetes syndrome*(DOID:11716) to be a subclass of *Diabetes mellitus*(DOID:9351), and based on this information we assume that any reference to, or mention of, *Prediabetes syndrome* is also a reference to *Diabetes mellitus* (with respect to DO).

There are several text mining systems designed for ontology concept recognition in text. These methods are either based on lexical methods and therefore applicable to a wide range of ontologies [6, 7] or they are domain-specific and rely on machine learning [8]. Text mining based-methods can also be used to automatically or semi-automatically construct and extend ontologies [9, 10]. For example, Lee et al. [11] focus on text mining of relations that are asserted in text between mentions of ontology classes that has been used to refine ontology classes in the Gene Ontology (GO) [12]. Text mining can also be used to suggest new subclasses and sibling classes in ontologies, for example Wächter and Schroeder [13] carried out a text mining based-system from different text sources which is used for extending OBO ontologies by semi-automatically generating terms, definitions and parent–child relations. Xiang et al. [14] have developed a pattern-based system for generating and annotating a large number of ontology terms, following ontology design patterns and providing logical axioms that may be added to an ontology. Recently, clustering based on statistical co-occurrence measures were also used to extend ontologies [15].

Here, we introduce a novel method relying on machine learning to identify whether a word used in text refers to a class that could be included in a particular ontology. Essentially, our method classifies terms to determine if they are usually mentioned in the same context as the labels and synonyms of classes in an ontology (which are used as seeds to train the classifier); this classifier can then be applied to unseen terms. Furthermore, our method can also be used to expand ontologies by suggesting terms that are mentioned within the same context as specific classes in an ontology.

We demonstrate the utility of our method in identifying words referring to diseases from DO in full text articles. We select the DO because the labels and synonyms of DO classes are relatively easy to detect in text and a large number of computational methods rely on access to a comprehensive disease ontology [16–19]. Our method achieves highly accurate (F-score > 90%) and robust results, is capable of recognizing multiple different classes including those defined formally through logical operators, and combines dictionary-based and context-based features; therefore, our method is also capable of finding new words that refer to a class. We manually evaluate the results and suggest several additions to the DO.

## Methods

### Building a disease dictionary

We built a dictionary from the labels and synonyms of classes in the Disease Ontology (DO), downloaded on 5 February 2018 from http://disease-ontology.org/downloads/. The dictionary consisted of 21,788 terms belonging to 6,831 distinct disease classes from DO. We utilized the dictionary with the Whatizit tool [20] and annotated the ontology class mentions along with their identifiers in approximately 1.6 million open access full-text articles from the Europe PMC database [21] (http://europepmc.org/ftp/archive/v.2017.06/) and generated a corpus annotated with mentions of classes in DO. We preprocessed the corpus by removing stop words such as “the”, “a”, and “is” as well as some punctuation characters.

### Generating context-based features

We use Word2Vec [22] to generate word embedding. Specifically, we use a skip-gram model which aims to find word representations that are useful for predicting the surrounding words in a given sentence or a document consisting of sequence of words; *w*_1_,*w*_2_,...,*w*_*K*_. The objective is to maximize the average log probability using the following formula:

1 $$ V({w}) = \frac{1}{K} \sum\limits_{k=1}^{K} \sum\limits_{-c\leq j\leq c; j\neq 0}^{K} log \; p(w_{K+j}|w_{K})  $$

where word vectors *V*(*w*) are computed by averaging over the number of words *K* and *c* is the size of the training context. We generated the word embedding by using the default parameter settings of the Word2Vec gensim implementation: vector size (dimensionality) of 100, window size 5, minimum occurrence count of 5, and we use a skip-gram (sg) model.

### Supervised training

We carried out a set of experiments to choose the optimal training algorithm to design our model. In our experiments we used default parameters for the training algorithms but different hidden layers for Artificial Neural Networks (ANNs) [23]. Our experiments show that the ANN model outperforms an SVM model [24] (see Additional file 1: Table 1 for full details), and our model performs best with 200 neurons in a single hidden layer (we tested a single hidden layer with a size of 10, 50, 100, and 200 neurons). We report results accordingly to a model with 200 neurons in the remainder of this work. In ANNs, multiple neurons are organized in layers. Typically, different layers perform different kinds of transformations on their inputs [25]. In our experiments, we used an ANN with an input layer of different sizes, a single hidden layer that uses a sigmoid activation function, and an output layer that differs based on the experiment. We train each classifier in a supervised manner, using 10-fold stratified cross-validation. Additionally, we report testing performance on an independent 20% testing set which we generated by randomly removing data points before training.

### Recognizing ontology classes in text

We used two approaches to recognize the mention of ontology classes in text. Our first approach relies solely on labels and synonyms of the classes within a given ontology *O* and can be used to determine whether a word refer to a class in *O*. We first obtain an ontology *O* in the Web Ontology Language (OWL) [26] format and extract a list of class labels and synonyms *L* from *O*; we further utilize a text corpus *T* as input to our method. Then, we generate word embeddings (i.e., vector-space encodings of the contexts in which a word occurs) for all words in our text corpus *T* and train a supervised machine learning model to classify whether a word refers to a class in *O* or not (using the *L*’s words as positive training instances and all others as negative instances).

**Fig. 1:**
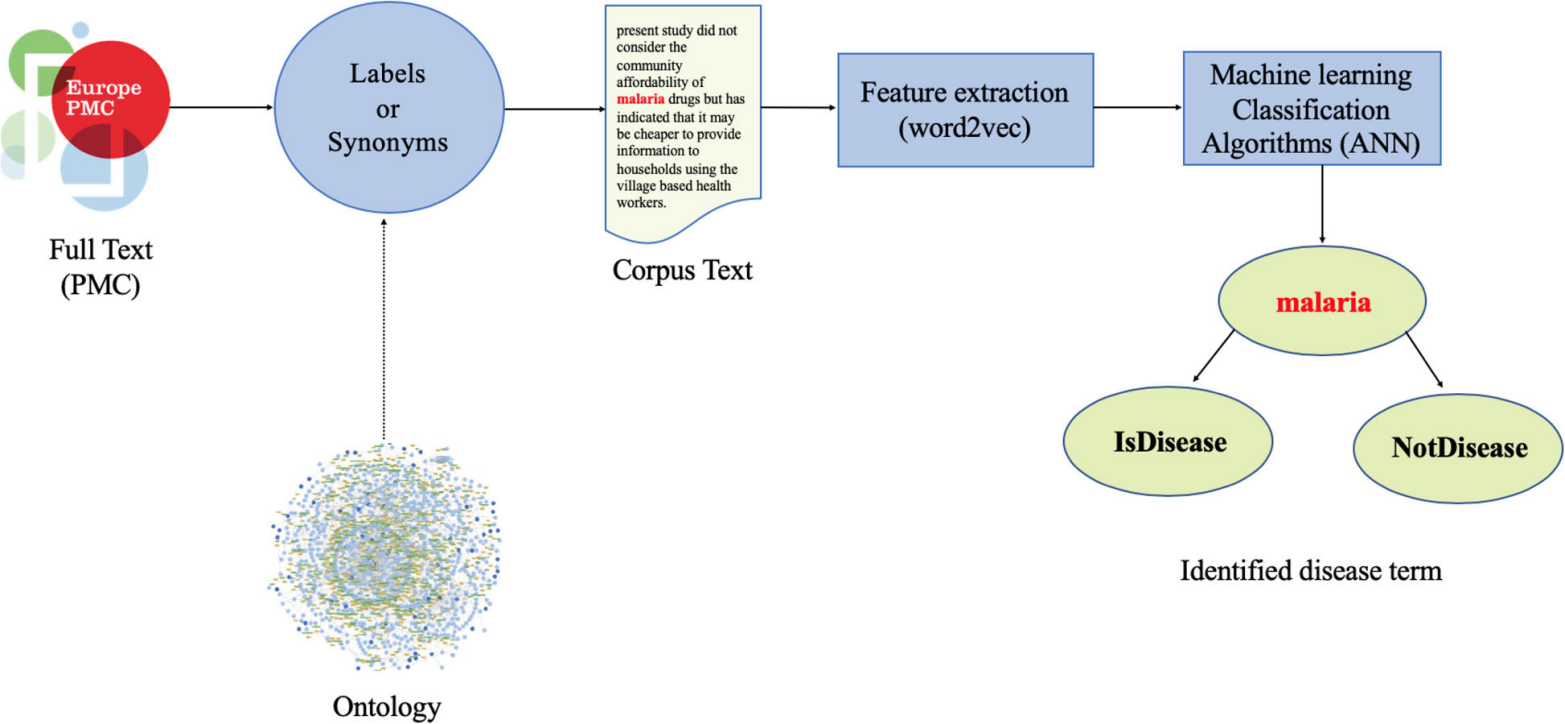
Label-based workflow. The workflow describes how words (in red) are classified as disease or “other”

Figure 1 illustrates the workflow of our first approach. Our method is generic and can, in principle, be applied to any ontology as long as the ontology provides labels (or synonyms), these labels can be identified in text, and the ontology from which the labels are extracted is more or less limited to a single domain. For example, reference ontologies in the OBO Foundry [27] are usually single domain ontologies and therefore suitable for our method. Ontologies that would not be suitable are application ontologies that cover multiple domains, such as the Experimental Factor Ontology (EFO) [28] (although our methods can be applied to parts of it). It is most useful to extend an existing ontology with new labels, synonyms, or classes.

In our second approach, we rely on annotations from the Whatizit tool [20] to identify the mention of ontology classes in text and determine their specific superclasses in an ontology. Our approach takes an ontology *O* in OWL format, a set of ontology classes *S*={*C*_1_,...,*C*_*n*_}, and a corpus of text *T* as inputs.

**Fig. 2:**
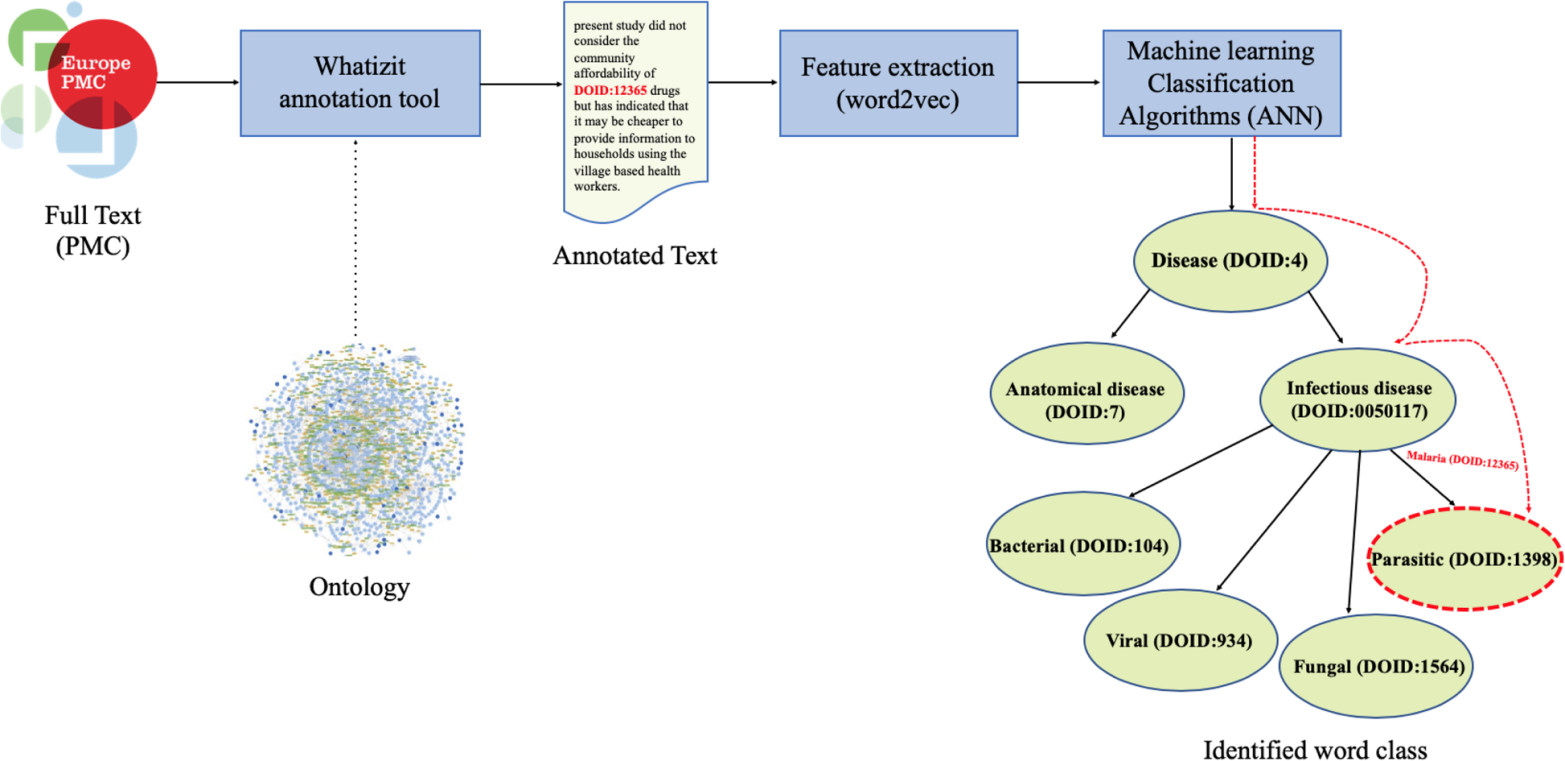
Annotation-based workflow. In this workflow, we first normalize the mentions of disease classes in the corpus and then apply Word2Vec to generate embeddings for classes, not merely words

This approach first uses Whatizit as a named entity recognition and normalization tool to normalize class labels and synonyms in text by replacing all mentions of a class with the class identifier (i.e., the class URI). We annotate 15,183 distinct terms using Whatizit; the total dictionary consists of 21,788 terms (derived from the labels and synonyms of classes in DO). We then train Word2Vec model that captures the context of the mention of the class and generates a vector space embedding for that class. Given such vector space embeddings for a set of classes in *O*, we use the vector space embeddings as input to a machine learning method that classifies whether another class appears in a similar context. We use this method to determine if a class should belong the superclass of *C* in *O*. Figure 2 illustrates the workflow of this approach.

The main difference between the two approaches is that the first approach broadly identifies terms or words that refer to classes within a domain (as defined by the sum of classes within an ontology) while the second approach can determine whether a term or word refers to a class that should appear as a subclass of a more specific ontology class. Both methods generate “seed” words in text and then use these seeds first to generate context-based features (through Word2Vec) and use these context-based features in a supervised machine learning classifier.

### Manual analysis process

We manually evaluate some of our findings. The manual evaluation is based on the medical expert knowledge of the evaluator who is a trained clinician, and supplemented by literature search to validate some findings or resolve conflicts. Mainly, results were confirmed by searching for review papers that characterize a condition. Overall, manual curation following the suggestions by our classifier took 10-15 min per sample (which included identifying related classes in the DO and drafting an explanation for cases which disagree with the DO).

## Results

### Broad classification of domain-specific terms: application to diseases

Our method is a workflow that can be used to identify whether a term or phrase commonly refers to a class that may be included in a domain-specific ontology as a label, synonym, or a new class. To achieve this goal, we use the existing labels and synonyms within a domain-ontology as “seeds” to train a machine learning classifier that determines whether a new term is sufficiently similar to an existing label or synonym and may therefore also be included in the ontology. We represent terms primarily by the context in which they occur within a large corpus of text; we use Word2Vec [22] for this purpose. We then train an Artificial Neural Network classifier in a supervised manner to distinguish between the terms already included within a domain ontology (and therefore expected to refer to a particular kind of phenomena) and randomly chosen terms not included in the ontology (and therefore most likely not referring to a phenomenon within the domain of the ontology).

We demonstrate our method using the Human Disease Ontology (DO) [5] and applying it to the terms occurring in a large corpus of full-text biomedical articles (see “Methods”). First, we tested whether our approach is capable of identifying words that refer to the *Disease* class (DOID:4), i.e., whether our method can detect terms that refer to a disease. We generated word embeddings for every disease terms and other words in our corpus of full-text articles.

**Fig. 3:**
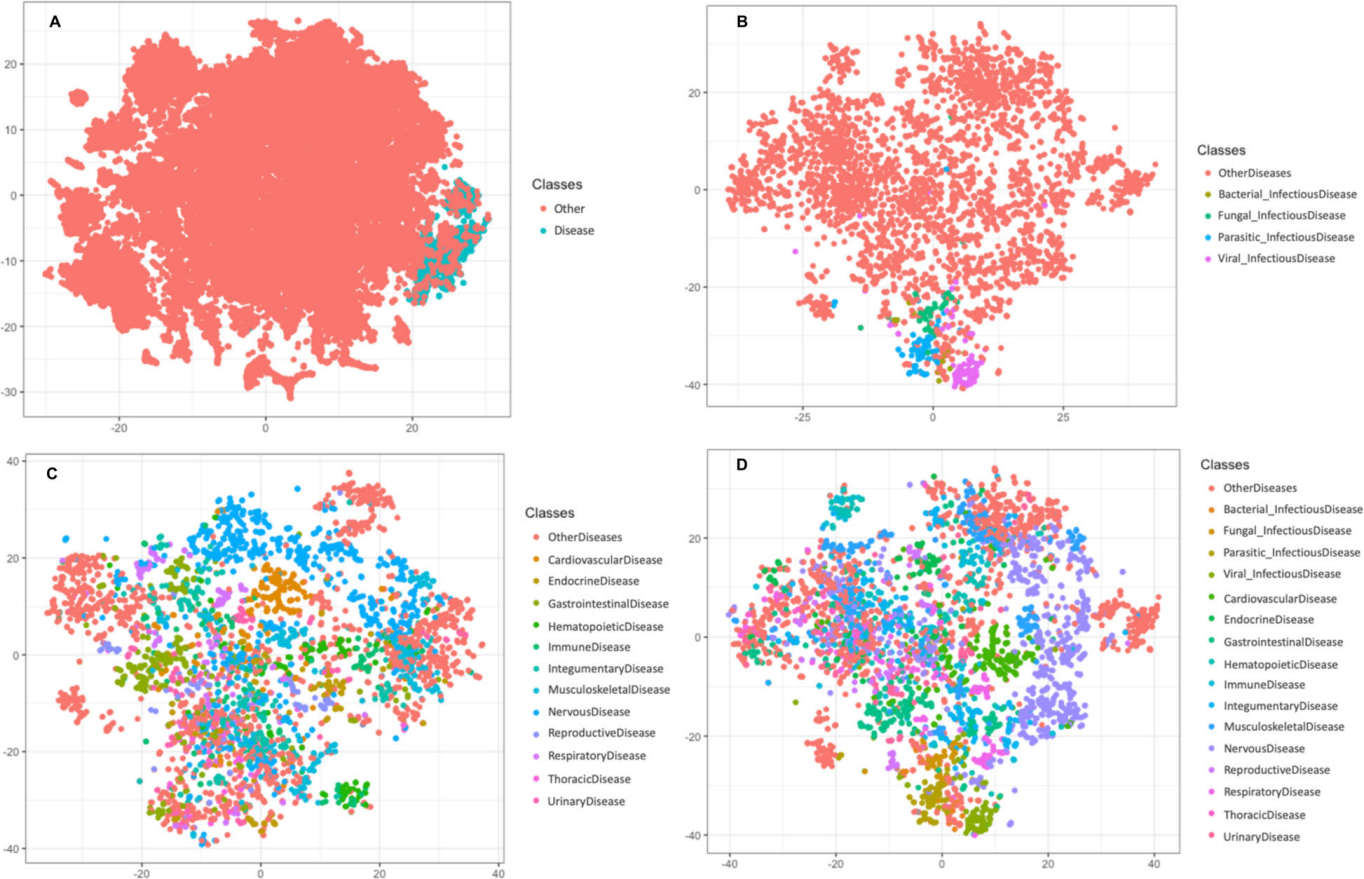
**a**) The visualization of the embeddings using the t-SNE for binary-classification task **b**) The visualization of the embeddings using the t-SNE for classifying infectious diseases. **c**) The visualization of the embeddings using the t-SNE for classifying anatomical diseases. **d**) The visualization of the embeddings using the t-SNE for classifying the combination of infectious and anatomical diseases

Figure 3 illustrates the distribution of the terms referring to a diseases in DO and other words mentioned in our corpus which do not belong to DO using the t-SNE dimensionality reduction [29]. We can see that the terms are clearly different and should be separable through a machine learning system.

**Fig. 4:**
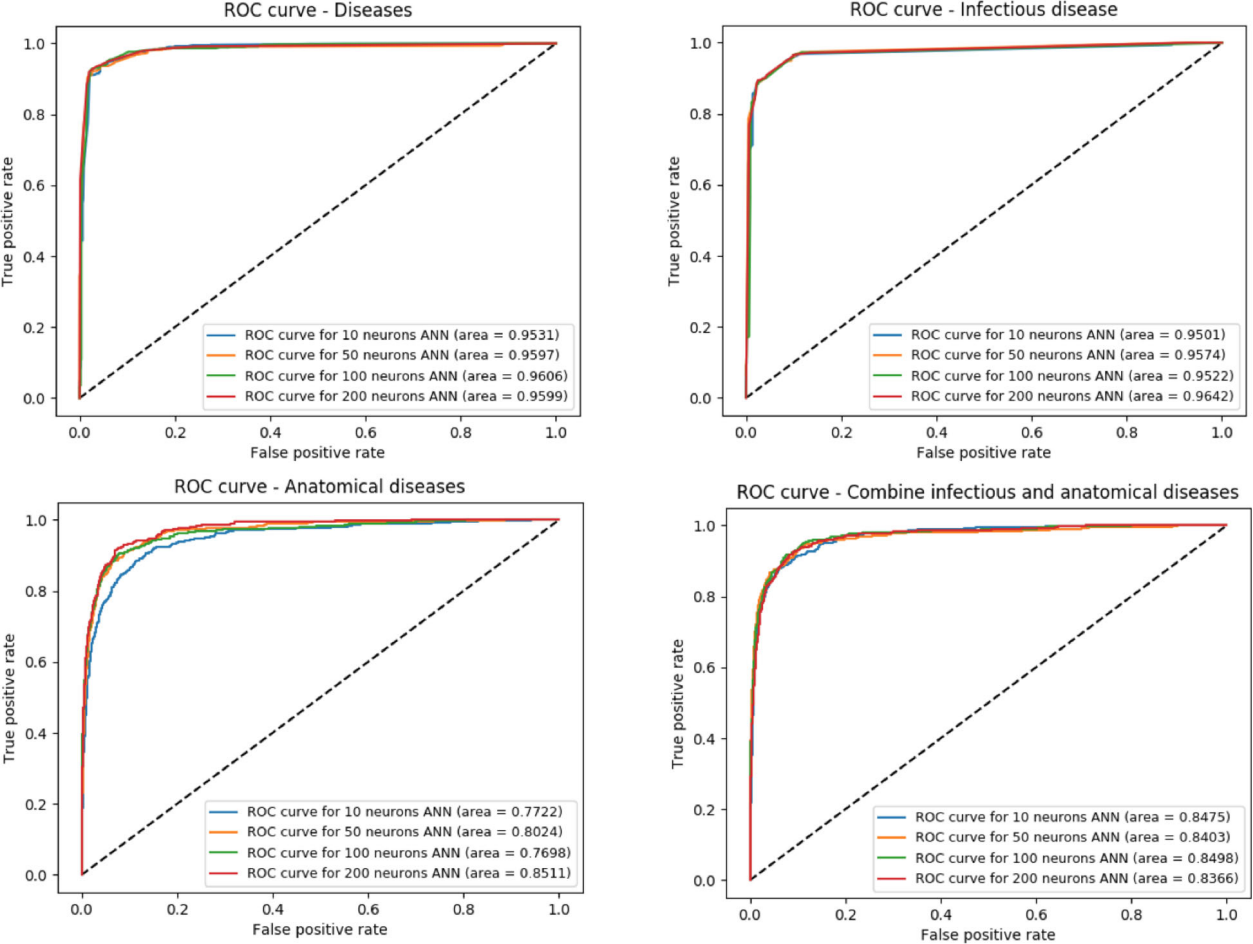
ROC curves for each experiment (Diseases, Infectious disease, Anatomical disease and a combination of Infectious disease + Anatomical disease)

Therefore, we trained a machine learning model to recognize whether a word refers to the disease or not using the word embeddings as input. We split the vector space embeddings into a training and testing dataset and consider all embeddings referring to disease as positive instances and all others as negatives. We do not apply any filtering before selecting the positive or negative samples. We randomly select negatives equal to the number of positives (7,932 positives and 7,932 negatives). We withhold 20% of randomly chosen positive and negative instances for testing, train a model on the remaining 80% through 10-fold cross validation, and report the performance results on the 20% test set. Evaluated on the testing set, we can distinguish between disease and non-disease terms with an F-score of 95% and AUC of 96% (see Table 1 and Figure 4).

**Table 1:** F-score and AUC for our four experiments using different hidden layer sizes

Classification	Hidden layer sizes	10		50		100		200	
	Number of classes	F-score	AUC	F-score	AUC	F-score	AUC	F-score	AUC
Diseases	2	94.65%	95.31%	94.83%	95.97%	**95.32%**	**96.06%**	94.49%	95.99%
Infectious disease	5	95.65%	95.01%	96.01%	95.74%	**95.43%**	95.22%	95.68%	**96.42%**
Anatomical disease	13	69.18%	77.22%	70.15%	80.24%	70.20%	76.98%	**72.00%**	**85.11%**
Infectious + anatomical diseases	17	71.07%	84.75%	**73.13%**	84.03%	72.61%	**84.98%**	72.67%	83.66%

The values in bold represent the highest AUC and F-score within each experiments

To better understand the source of errors and whether our approach can be used to reliably extend ontologies (either with additional labels and synonyms, or new classes), we performed a manual analysis on a set of 20 false positive samples out of 197 which are not the label or synonym of a disease class DO but are classified as disease by our classifier (see Table 2). We found that the majority of the 20 false positive samples refer to either diseases or phenotypes (where phenotypes are the observable characteristics of an organism that may occur manifestations, or signs and symptoms, of a disease, but do not constitute a disease on its own). For example, *Aphthosis* is a prediction of our method which refers to a human disorder that is not currently in the DO; the majority of false positives are disease-related terms that do not explicitly refer to a disease. For example, we predicted *mal-absorption* as a disease term which may refer to a phenotype in some contexts. Our findings indicate that an ANN classifier can identify known terms referring to diseases, and can further suggest novel terms which may prove useful for ontology development and extension.

**Table 2:** Manually analyzed disease terms predicted as disease

Term	Manual analysis result	Explanation for the suggested diseases
FACTO	other	-
leucoencephalopathy	other	-
**Aphthosis**	Disease	A disease refers to a condition with repetitive mucosal ulcers [30, 31].
Desmoid	other	-
metapneumovirus	other	-
**Tracheobronchomalacia**	Disease	A rare condition with abnormal flaccidity of both the trachea and the bronchi which results in possibility of narrowing or collapse of the airway [32–34].
**RESLES**	Disease	A rare condition characterized by transient lesions in the central part of the splenium of the corpus callosum (SCC), followed by complete reversibility on follow-up magnetic resonance imaging (MRI) after a variable period. It coincides with different diseases [35, 36].
mal-absorption	other	-
acroparesthesias	other	-
limb-shaking	other	-
**pineocytomas**	Disease	A rare disease that has an Orphanet ID: ORPHA:251912. It is one of the pineal parenchymal tumors and is considered the least aggressive one [37, 38].
hypomineralisation	other	-
**neurognathostomiasis**	Disease	It is a severe form of human gnathostomiasis, DOID:11379, which can lead to disease and death, it involves the nervous system [39–41].
Metastasis	other	-
**myelomatosis**	Disease	A type of cancer that begins in plasma cells that produce antibodies. It could be one of the synonyms of multiple myeloma DOID:9538 [42, 43].
**AMRF**	Disease	An OMIM disease, OMIM:254900 [44].
arthralgia	other	-
**fibrodentinoma**	Disease	Fibrodentinoma is a benign odontogenic tumor that occurs in children and young adults. The disease name usually is represented as “Ameloblastic Fibrodentinoma” [45, 46].
infantile-ataxia	other	-
knowlesi	other	-

The terms in bold represent the correctly validated terms (by a clinician) that classified as diseases terms using our method (in Diseases classification experiment).

### Fine-grained classification: distinguishing between groups of diseases

As our method showed capability to identify terms referring to a disease, we next tested whether our method can also distinguish between different types of diseases. For this purpose, we used the embeddings generated from a pre-processed corpus in which we normalize all mentions of a disease in our corpus using Whatizit tool. The disease dictionary that we utilized with Whatizit includes a total of 21,788 terms (labels and synonyms) from DO. We found that 15,183 of these 21,788 terms appeared in our corpus and we generate an embedding vector for each of them. We then first trained a neural network model to recognize whether a disease-term refers to the *Infectious Disease*(DOID:0050117) class or not, and furthermore whether our method is able to distinguish between the four different types of infectious disease in DO (i.e., bacterial, fungal, parasitic, or viral infectious disease). As training data, we used the word embeddings generated for DO classes, and we used the Elk reasoner to split them into four types of infectious diseases, and an additional class for diseases that are not a subclass of *Infectious Disease* in DO. We randomly select 20% of the disease in DO as validation set and train the neural network classifier using 10-fold cross-validation on the remaining 80% to separate diseases into one of the five classes (non-infectious, bacterial, fungal, parasitic and viral infections). Table 1 shows the performance achieved on the validation set. While the performance is less than predicting whether a term refers to a disease, our classifier can distinguish between specific disease classes.

We manually analyzed a set of 20 false positive samples out of 38 which are not a subclass of *Infectious disease* in the DO but are classified as infectious by our classifier (see Table 3). We found that 7 of these 20 cases can be suggested to be subclasses of the specific infectious disease they have been classified with but do not have a subclass relation asserted or inferred in DO. For example, the term *syphilitic meningitis* (DOID:10073) is a disease that our method classify as a bacterial infectious disease but it is not classified as infectious in the DO.

**Table 3:** Sample of manually analyzed disease terms predicted as infectious disease

Disease terms	Ontology class assigned by ANN	Manual analysis result	Suggested additional classification	DOID	Explanation
Pelizaeus-Merzbacher disease	Viral infectious disease	Non-infectious (inherited disorder)	-	-	-
**Kaposi’s sarcoma**	Viral infectious disease	Viral infectious disease	herpes simplex	DOID:8566	The disease is caused by Human herpesvirus 8 which is Herpesviridae infection.
**maxillary sinusitis**	Bacterial infectious disease	Bacterial infectious disease (usually start viral and progress to either bacterial or fungal)	-	-	It is an infection in the maxillary sinuses which could be due to different etiology, one of them is bacterial [47].
keratosis follicularis	Bacterial infectious disease	Non-infectious (genetic disease)	-	-	-
chronic rheumatic pericarditis	Viral infectious disease	The condition is triggered by autoimmune reaction to infection, mainly group A streptococci.	-	-	-
**gastroparesis**	Viral infectious disease	In most cases the nerve is damaged by diabetes or surgery, however, a viral infection might be a cause	-	-	A condition in which the stomach suffers from paresis that affects the food movement to the small intestine [48, 49].
osmotic diarrhea	Bacterial infectious disease	symptom	-	-	-
familial cold autoinflammatory syndrome	Viral infectious disease	Non-infectious (inherited disease)	-	-	-
**angular cheilitis**	Fungal infectious disease	Etiology is controversial, most commonly fungal or bacterial.	-	-	Ambiguous.
Binder syndrome	Viral infectious disease	Congenital disease	-	-	-
hypohidrosis	Bacterial infectious disease	Multi-causal	-	-	-
Sjogren’s syndrome	Viral infectious disease	autoimmune disease	-	-	-
**median rhomboid glossitis**	Fungal infectious disease	Etiology is controversial, however it is considered as a variant of orallesion associated with candida infection [50].	-	-	Ambiguous.
Goodpasture syndrome	Viral infectious disease	autoimmune disease	-	-	-
**syphilitic meningitis**	Bacterial infectious disease	Bacterial infectious disease	syphilis	DOID:4166	Considering the same concept of etiology, both diseases are caused by bacterial infection (Treponema pallidum).
acute diarrhea	Viral infectious disease	symptom	-	-	-
WHIM syndrome	Bacterial infectious disease	Congenital disease	-	-	-
erythrasma	Fungal infectious disease	Bacterial infection disease	-	-	-
chronic wasting disease	Parasitic infectious disease	Neurodegenerative disorder	-	-	-
**scarlet fever**	Bacterial infectious disease	Bacterial infectious disease	rheumatic fever	DOID:1586	The disease is caused by Group A bacteria of the genus Streptococcus, same causative agent for Rheumatic fever.

The terms in bold represent the correctly validated terms (by a clinician) that classified as infectious diseases terms using our method (in Infectious disease classification experiment).

Moreover, to test the strength of our method to distinguish between disease classes, we further trained a neural network model to distinguish between the 12 different subclasses of *Disease of anatomical entity*(DOID:7), as well as an additional class for diseases not classified as subclasses of *Disease of anatomical entity*. We used the same method to split the classes in training and test set as before. Results are shown in Table 1 and demonstrate that our method can also be useful to classify diseases in their anatomical sub-systems.

We manually analyzed a set of 20 false positive samples out of 127 which are not a subclass of *Anatomical disease* in the DO but are classified as being a subclass of a particular anatomical system disease by our classifier (see Table 4). We found that 12 of the 20 false positives can be suggested to be subclasses of the specific anatomical system disease they have been classified with but do not have such a subclass relation asserted or inferred in DO. For example, we classify *Narcolepsy* (DOID:8986) as a *Nervous system anatomical disease*, and this may be added as a new subclass axiom to DO.

**Table 4:** Sample of manually analyzed disease terms classified as affecting particular anatomical systems (*Continued*)

Disease terms	Ontology class	Ontology class assigned by ANN	Manual analysis result	Suggested additional classification	DOID	Explanation
Timothy syndrome	genetic disease	cardiovascular system disease	Cannot specify (affect multiple parts)	-	-	-
Familial periodic paralysis	disease of metabolism	cardiovascular system disease	musculoskeletal system disease	-	-	-
**Hyperprolactinemia**	disease of metabolism	endocrine system disease	endocrine system disease	pituitary gland disease	DOID:53	The pituitary gland is the endocrine gland responsible for secreting prolactin.
Angiokeratoma circumscriptum	disease of cellular proliferation	gastrointestinal system disease	cardiovascular system disease	-	-	-
**Zollinger-Ellison syndrome**	syndrome	gastrointestinal system disease	gastrointestinal system disease	peptic ulcer disease	DOID:750	It is a disease that affects either pancreas, duodenum, or both of them. Both organs are pats of the GIT system. The disease pathology is mainly excessive gastrin secretion with subsequent peptic ulcers.
**Polycystic liver disease**	genetic disease	gastrointestinal system disease	gastrointestinal system disease	liver disease	DOID:409	It is a genetic disorder that affects primarily the liver.
**Bilirubin metabolic disorder**	disease of metabolism	hematopoietic system disease	hematopoietic system disease	kernicterus due to isoimmunization	DOID:12043	Bilirubin disorder could be a result of blood pathology, same as for the mentioned classification DOID:12043.
**Alpha thalassemia**	genetic disease	hematopoietic system disease	hematopoietic system disease	hemoglobinopathy	DOID:2860	The disease is mainly a hemoglobin disorder with hematological phenotypes.
Kabuki syndrome	syndrome	immune system disease	Not anatomical - multisystems	-	-	-
Amyloidosis	disease of metabolism	immune system disease	Not anatomical - multisystems	-	-	-
Fatty liver disease	disease of metabolism	musculoskeletal system disease	gastrointestinal system disease	-	-	-
Renal-hepatic-pancreatic dysplasia	physical disorder	musculoskeletal system disease	Cannot specify (affect multiple parts)	-	-	-
**Radioulnar synostosis**	physical disorder	musculoskeletal system disease	musculoskeletal system disease	bone development disease/Synostosis	DOID:0080006/ DOID:11971	There is already an entity in the DO for synostosis under bone development disease.
**Hypophosphatasia**	genetic disease	musculoskeletal system disease	musculoskeletal system disease	bone remodeling disease	DOID:0080005	We could suggest an additional classification based on the main affected system. Our suggestive classification is musculoskeletal since
						the disease is mainly affecting mineralization of the bone with phenotypes similar to those of Rickets DOID:10609.
**Narcolepsy**	disease of mental health	nervous system disease	nervous system disease	*	*	*
**Aceruloplasminemia**	disease of metabolism	nervous system disease	nervous system disease	neurodegeneration with brain iron accumulation	DOID:0110734	The disease main pathophysiology is either the absence or dysfunction of ceruloplasmin with subsequent iron accumulation in various organ, mainly the brain.
Glomangiomatosis	disease of cellular proliferation	nervous system disease	cardiovascular system disease	-	-	-
**Deafness-dystonia-optic neuronopathy syndrome**	disease of metabolism	nervous system disease	nervous system disease	nervous system disease; since it covers many subclasses to which we can map many aspects of this disease	DOID:863	The disease’s phenotypes reflect neurological affection of multiple parts in the nervous system.
**Trophoblastic neoplasm**	disease of cellular proliferation	reproductive system disease	reproductive system disease	Female reproductive organ cancer	DOID:120	The term refers to the group of malignant neoplasms that consist of abnormal proliferation of trophoblastic tissues similar to choriocarcinoma DOID:3596 and gestational trophoblastic neoplasia DOID:3590.
**Cryptorchidism**	physical disorder	reproductive system disease	reproductive system disease	testicular disease	DOID:2519	The term refers to undescended testicle.

^*^Nacrolepsy: is classified as a sleep disorder which is correct, however, the class itself is a subclass to mental disorders. Since there are some neurological disorders that have shown a strong association with sleep disorder such as: neurodenegrative disorders such as tauopathy which involve Alzheimer’s diseases (DOID:10652) [51], synucleinopathy which involve Parkinsonism (DOID:14330) [52], and Genetic neurodegenerative disorders such as Machado-Joseph disease (DOID:1440) [53] or Huntington’s disease (DOID:12858) [54]. We suggest a new classification in which sleep disorders may also be a subclass of nervous system diseases (neurodegenerative disorder) [55] The terms in bold represent the correctly validated terms (by a clinician) that classified as anatomical diseases terms using our method (in Anatomical disease classification experiment).

As it is often inconvenient to train separate classifiers, we also combined both tasks and trained a multi-class classifier to classify disease classes either as infectious or anatomical, or as other disease. We evaluate the performance of this combined model (see Table 1), and our machine learning system achieves an AUC up to 84% (see Figure 4). These results demonstrate it may be possible to identify new subclasses, although the performance drops when we increase the complexity of the classification problem by distinguishing between more subclasses.

## Discussion

We developed a method to automatically expand ontologies in the biomedical domain with new classes, synonyms, or axioms. We demonstrate the utility of our approach on the DO [5] which is widely used in biomedical research [56]. As case studies, we focused on two high-level classes in the DO: *Infectious Diseases* and *Anatomical Diseases*. We have evaluated our method both using common performance measures in machine learning as well as through manually investigating some of the predicted false positives.

When applying our method to the DO, our false positive predictions often include phenotypes or, in some cases, pathogens. It is well-established that it is challenging to distinguish between diseases and phenotypes in literature [57–59], as evidenced by the large overlap between disease ontologies and phenotype ontologies [19]. Similarly, diseases and pathogens can often have very similar names [60, 61], thereby making it challenging to distinguish between them. While a disease is defined as the structural or functional disorder that usually results in symptoms, signs and physical or chemical changes, phenotype refers to observable characteristics of an organism and may be a part of a disease manifestation. Phenotype terms cover disease symptoms, signs and the investigational results that might be related to that disease. Some phenotypic terms are more diverse; for example, congenital hemolytic anemia is a form of hemolytic anemia with congenital onset. The term is included in both the Human Phenotype Ontology (HP) (HP:0004804) and disease ontology (DOID:589). From a clinical point of view, it could be a type of disease under the umbrella of hemolytic disorders with a congenital onset; however, congenital hemolytic anemia may also be a phenotype for certain diseases. For this reason, deciding on some terms to be identified either as phenotypes or diseases can be complex, challenging, and context-dependent.

Another limitation of our method is the use of the Whatizit tool [20] to detect and normalize mentions of ontology classes in text. In our first use-case – the extension of ontologies with new labels and synonyms – we classify terms that occur in text without relying on any prior text processing which has some drawbacks such as considering a word as disease name within a general context. We use Whatizit for our second use-case – the detection of subclass axioms – while the performance of Whatizit is less than domain- and task-specific named entity recognition and normalization tools [62], Whatizit’s key advantage is that it is a lexical, rule-based method that does not require any training and is able to recognize multi-word terms. Whatizit can therefore be applied to a wide range of ontologies without the need to generate a training dataset. To evaluate the performance of Whatizit, we tested it on the NCBI disease corpus [16] using their test set containing 100 abstracts. In our evaluation, Whatizit has a precision of 75% and recall of 15% and an F-score of 26% with an accuracy of 90% (see Additional file 2). One of the reasons for the low recall is the number of diseases which are included in the Medical Subject Headings (MeSH) [63] or the Online Mendelian Inheritance in Man (OMIM) [64] vocabulary but not in DO. Furthermore, Whatizit ignores many disease abbreviations since they are not included in DO (and therefore in the vocabulary used by Whatizit).

## Conclusions

We presented a general method for semi-automatically extending ontologies with new labels, synonyms, classes, or some general subclass axioms. Our approach is based on machine learning algorithms utilizing vector representation of the ontology classes generated from full text articles. We demonstrated the utility of our approach on the Human Disease Ontology (DO), specifically by finding new candidate classes, labels, and synonyms to add to DO such as *Aphthosis*, and by identifying new axioms that relate disease classes to their infectious agent or anatomical systems. Our method can help to improve the quality and coverage of ontologies in the ontology development process by automatically suggesting terms to include (either as labels of new classes or synonyms of existing classes) and suggesting missing subclass axioms. In the future, we plan to expand our study to other ontologies and to defined classes to further analyze its robustness.

## Supplementary information

**Additional file 1** Different conducted experiments based on different classification tasks.

**Additional file 2** The evaluation of analyzing NCBI abstracts annotated using Whatizit tool.

## Data Availability

All source code developed for this study is available from https://github.com/bio-ontology-research-group/ontology-extension.

## Abbreviations

ANNs: Artificial neural networks
AUC: Area under curve
DO: Disease ontology
EFO: Experimental factor ontology
GO: Gene ontology
HP: human phenotype ontology
MeSH: Medical subject headings
OMIM: Online mendelian inheritance in man
OWL: Web ontology language
ROC: Receiver operating characteristic
Sg: Skip-gram
