# Supplementary material for: Combining lexical and context features for automatic ontology extension
Source: J Biomed Semantics. 2020 Jan 13;11:1. doi: 10.1186/s13326-019-0218-0 (PMC6958746; doi:10.1186/s13326-019-0218-0)
Supplement: Supplementary file 1 — Additional file 1 Different conducted experiments based on different classification tasks. [file 13326_2019_218_MOESM1_ESM.pdf]

Supplementary materials: Combining lexical and context features for automatic ontology extension

Table 1: Prediction performances of using linear kernel support vector machine (SVM) algorithm evaluated by AUC and F-score.

Classification	Number of classes	Linear SVM	
		F-score	AUC
Diseases	2	92.84%	93.72%
Infectious disease	5	92.82%	93.49%
Anatomical disease	13	65.35%	72.76%
Infectious + anatomical diseases	17	67.76%	76.51%

Table 2: Prediction performances of using Artificial neural network (ANN) using different hidden layer sizes evaluated by accuracy. Accuracy is reported on a 20% test set randomly chosen from the positives and negatives.

Classification	Number of positive	Number of negative	Hidden layer sizes	10	50	100	200
			Number of classes	Accuracy			
Diseases	80,086	80,086	2	97.43%	98.62%	98.50%	94.77%
Infectious disease	379	3,754	5	96.35%	96.55%	96.78%	96.48%
Anatomical disease	2,595	1,538	13	56.30%	60.99%	59.58%	60.26%
Infectious + anatomical diseases	2,974	1,159	17	59.44%	61.31%	60.78%	62.33%
